# Assessing Image Quality in Multiplexed Sensitivity-Encoding Diffusion-Weighted Imaging with Deep Learning-Based Reconstruction in Bladder MRI

**DOI:** 10.3390/diagnostics15050595

**Published:** 2025-02-28

**Authors:** Seung Ha Cha, Yeo Eun Han, Na Yeon Han, Min Ju Kim, Beom Jin Park, Ki Choon Sim, Deuk Jae Sung, Seulki Yoo, Patricia Lan, Arnaud Guidon

**Affiliations:** 1 Department of Radiology, Korea University Anam Hospital, Korea University College of Medicine, 73 Goryeodae-ro, Seongbuk-gu, Seoul 02841, Republic of Korea; seunghacha@gmail.com (S.H.C.); yeonny0714@korea.ac.kr (Y.E.H.); dr.minjukim@gmail.com (M.J.K.); radiolbj226@gmail.com (B.J.P.); ha2sky@hanmail.net (K.C.S.); urorad@korea.ac.kr (D.J.S.); 2 GE Healthcare, 416 Hangang-daero, Seoul 04637, Republic of Korea; seulki.yoo@gehealthcare.com; 3 MR Collaborations & Clinical Solutions, GE HealthCare, Menlo Park, CA 94025, USA; 4 MR Collaborations & Clinical Solutions, GE Healthcare, Boston, MA 02142, USA

**Keywords:** bladder MRI, diffusion-weighted imaging (DWI), deep learning reconstruction, image quality

## Abstract

**Background/Objectives:** This study compared the image quality of conventional multiplexed sensitivity-encoding diffusion-weighted imaging (MUSE-DWI) and deep learning MUSE-DWI with that of vendor-specific deep learning (DL) reconstruction applied to bladder MRI. **Methods:** This retrospective study included 57 patients with a visible bladder mass. DWI images were reconstructed using a vendor-provided DL algorithm (AIR^TM^ Recon DL; GE Healthcare)—a CNN-based algorithm that reduces noise and enhances image quality—applied here as a prototype for MUSE-DWI. Two radiologists independently assessed qualitative features using a 4-point scale. For the quantitative analysis, signal-to-noise ratio (SNR), contrast-to-noise ratio (CNR), signal intensity ratio (SIR), and apparent diffusion coefficient (ADC) of the bladder lesions were recorded by two radiologists. The weighted kappa test and intraclass correlation were used to evaluate the interobserver agreement in the qualitative and quantitative analyses, respectively. Wilcoxon signed-rank test was used to compare the image quality of the two sequences. **Results:** DL MUSE-DWI demonstrated significantly improved qualitative image quality, with superior sharpness and lesion conspicuity. There were no significant differences in the distortion or artifacts. The qualitative analysis of the images by the two radiologists was in good to excellent agreement (κ ≥ 0.61). Quantitative analysis revealed higher SNR, CNR, and SIR in DL MUSE-DWI than in MUSE-DWI. The ADC values were significantly higher in DL MUSE-DWI. Interobserver agreement was poor (ICC ≤ 0.32) for SNR and CNR and excellent (ICC ≥ 0.85) for SIR and ADC values in both DL MUSE-DWI and MUSE-DWI. **Conclusions:** DL MUSE-DWI significantly enhanced the image quality in terms of lesion sharpness, conspicuity, SNR, CNR, and SIR, making it a promising tool for clinical imaging.

## 1. Introduction

Bladder cancer is a significant health concern, being the sixth most common malignancy in men in 2022, with an age-standardized incidence rate of 9.3 per 100,000 men [1]. Bladder cancer most commonly presents with gross hematuria, but it can also be identified through microscopic hematuria, irritative voiding symptoms, or as an incidental finding on imaging [2]. Transurethral resection of bladder tumors (TURBT) remains a fundamental procedure for the diagnosis and staging of bladder cancer. However, TURBT has inherent limitations, as residual tumor tissue is often observed after resection, which may result in understaging. Magnetic resonance imaging (MRI) is increasingly being recognized as a valuable tool for the assessment of bladder tumors. With recent advancements in imaging technology, MRI, with its high multiparametric capability, has shown superior accuracy compared to CT, particularly in differentiating between the non-muscle-invasive and muscle-invasive forms of bladder cancer [3].

Diffusion-weighted imaging (DWI) is crucial for the evaluation of bladder cancer using multiparametric MRI and significantly contributes to the detection, staging, and grading of the disease [4,5,6]. The Vesical Imaging Reporting and Data System (VI-RADS) was developed in 2018 to standardize the imaging and reporting of bladder cancer using multiparametric MRI [7]. VI-RADS scoring serves as an excellent tool for preoperative T-staging of high-risk bladder cancer patients [8], and DWI plays a central role in assessing muscular invasion alongside T2-weighted and dynamic contrast-enhanced images [4]. Additionally, by enhancing the visibility of small lesions that may be missed on T2-weighted images, DWI increases overall tumor detectability [4]. Furthermore, the apparent diffusion coefficient (ADC) values derived from DWI contribute to the evaluation of tumor cellularity and have been shown to correlate with the histological grade and cell cycle regulators such as p53, p21, and Ki-67 in bladder cancer, serving as a predictor of aggressiveness [9,10,11]. Moreover, DWI can more effectively distinguish residual or recurrent tumors from the acute inflammatory changes commonly encountered after TURBT, thereby aiding in the evaluation of recurrent tumors [12].

However, conventional DWI obtained using single-shot echo-planar imaging (SS-EPI) has limitations for assessing bladder cancer. The image quality of SS-EPI is often impaired by significant distortion caused by susceptibility artifacts from gases in the small intestine and colon [13]. These susceptibility artifacts also increase with higher magnetic field strength, making it even more challenging to obtain clear images [13]. Additionally, conventional DWI techniques often suffer from poor spatial resolution and insufficient tissue contrast, and thus may miss small bladder cancers [14].

Recently, advanced imaging techniques, such as multiplexed sensitivity-encoding diffusion-weighted imaging (MUSE-DWI), have been introduced to enhance image quality in DWI. MUSE-DWI effectively reduces geometric distortions, enhances spatial resolution, and increases the signal-to-noise ratio (SNR), thereby providing superior image quality compared with conventional SS-EPI methods. It achieves these improvements by interleaving k-space partitioning along the phase-encoding direction across multiple excitations. This multiplexed approach accelerates the filling of k-space data and simultaneously corrects phase errors induced by organ motion [15,16,17]. However, despite its notable advantages, MUSE-DWI has prolonged acquisition times, which limits its broad clinical applicability. To address this limitation, vendor-specific DL reconstruction, AIR™ Recon DL (ARDL), has been integrated into MUSE-DWI. ARDL utilizes deep neural networks to optimize MR reconstruction, improving sharpness and SNR, thus allowing for a reduction in scan time while maintaining image quality.

This retrospective study aimed to compare the image quality of conventional MUSE-DWI with that of DL MUSE-DWI with vendor-specific DL reconstruction applied to bladder MRI. By evaluating the image quality of these two sequences, we aimed to determine the potential advantages of incorporating ARDL into routine bladder imaging protocols.

## 2. Materials and Methods

### 2.1. Patients

This retrospective study was approved by the Institutional Review Board (protocol code: 2025AN0018), and the requirement for informed consent was waived. From November 2023 to June 2024, 59 patients underwent bladder MRI, including the MUSE-DWI sequence. Among these, 2 patients without visible masses were excluded, resulting in the final cohort of 57 patients enrolled in this study. None of the 57 patients had severe artifacts or magnetic susceptibility artifacts related to metallic devices in the pelvis.

### 2.2. MRI Data Acquisition

All patients underwent bladder MRI using a 3.0 T MRI scanner (SIGNA^TM^ Premier, GE Healthcare, Waukesha, WI, USA) equipped with a flexible phased-array 21-channel coil (AIR^TM^ multipurpose coil, GE HealthCare). Patients held their urine for at least 1 h before the examination, and antispasmodic agents were administered to minimize motion artifacts. This study utilized a prototype version of ARDL on MUSE-DWI, in which conventional MUSE-DWI was initially acquired without ARDL, followed by retrospective reconstruction to generate DL MUSE-DWI with ARDL. By employing both reconstruction methods, we could directly compare the performance and efficacy of the deep learning-enhanced pipeline with those of the standard approach. The sequence parameters were set as follows: repetition time (TR) = 6800 ms, minimum echo time (TE) = 56 ms, slice thickness = 4 mm, slice spacing = 0.4 mm, number of slices = 38, matrix size = 128 × 128, field of view = 250 × 250 mm, b-values = 0, 600, and 1000 s/mm^2^, number of excitations = 2 (b600) and 4 (b1000), and acquisition time = 4 min 46 s.

### 2.3. Deep Learning Reconstruction Algorithm

The deep learning reconstruction algorithm used in this study was a vendor-provided prototype version of the AIR^TM^ Recon DL for the MUSE-DWI, which is not yet commercially available but was clinically evaluated here for its potential to generate enhanced-quality MUSE-DWI images [18]. This ARDL model was trained using a deep convolution neural network (CNN) with a supervised learning approach on a large dataset of approximately 4 million image pairs, each representing conventional and near-perfect MR images. This approach enabled the generation of high-fidelity images from raw k-space data by minimizing truncation artifacts, improving edge sharpness, and reducing noise. The ARDL model was added to the standard vendor pipeline to ensure MUSE reconstruction steps align with the non-deep learning-based approach. Among the available user-specific denoising levels (low, medium, or high), the ‘high’ DL strength was chosen for reconstructing MR images offline from the raw k-space data.

### 2.4. Qualitative Image Analysis

All images were transferred to a GE post-processing workstation (Advantage Workstation Server 3.2 Ext 4.8, 2022; GE Healthcare). DWI images were processed using the READYView software package on this workstation to generate ADC maps for both MUSE-DWI and DL MUSE-DWI based on b0 and b1000 images. Image analysis was performed by two radiologists with 3 and 16 years of experience in interpreting bladder MRI. Axial DWI images with a b-value of 1000 s/mm^2^ were reviewed by two reviewers in two separate sets (MUSE-DWI and DL MUSE-DWI) 2 weeks apart. The reviewers were blinded to the DWI sequence used. During the qualitative analysis, the two reviewers independently rated the sharpness, distortion, artifacts, and lesion conspicuity of MUSE-DWI and DL MUSE-DWI on a 4-point scale. The detailed scoring criteria were as follows: sharpness (1: unclear; 2: slightly unclear; 3: moderate; 4: clear), distortion (1: severe distortion; 2: moderate distortion; 3: slight distortion; 4: no distortion), artifacts (1: severe and non-diagnostic quality; 2: serious and possibly affecting the local diagnosis; 3: slight and not affecting the diagnosis; 4: no artifacts), and lesion conspicuity (1: lesion cannot be identified; 2. most of the lesion contours were not clear; 3, good lesion conspicuity with a small part of the outline not clear; 4, excellent lesion conspicuity with a clear outline).

### 2.5. Quantitative Image Analysis

For the quantitative analysis, two reviewers independently drew regions of interest (ROIs) on the axial MUSE-DWI b1000 sequence for the bladder tumor, gluteus maximus muscle, and background air. Circular ROIs were drawn at the homogeneous part of the bladder tumor with high signal intensity to minimize variability caused by tumor heterogeneity, avoiding parts with tumor necrosis, hemorrhage, and the tumor stalk. There was no consensus on ROI placement between the two radiologists. The tumor with the largest diameter was selected when multiple tumors were present. The area of the ROI was 3~1331 mm^2^ for bladder tumors, depending on the size of the tumor, with larger tumors allowing for larger ROIs and small tumors requiring smaller ROIs. The area of the ROI was set to 100 mm^2^ for the gluteus maximus muscle and the background air. All ROIs were copied and pasted onto the DL MUSE diffusion image and corresponding ADC maps at the same location and slice. The signal intensity (SI), standard deviation (SD), and ADC value of the bladder tumor, SI and SD of the gluteus maximus muscle, and SD of the background air were recorded. The SNR, contrast-to-noise ratio (CNR), and signal intensity ratio (SIR) were calculated using the following formulas, where $S_{lesion}$ is the mean SI of the bladder tumor, $S_{psoas}$ is the mean SI of the psoas muscle, ${SD}_{background}$ is the SD of the background noise, ${SD}_{lesion}$ is the SD of the tumor SI, and ${SD}_{psoas}$ is the SD of the psoas muscle SI:

$$SNR=\frac{S_{lesion}}{{SD}_{background}}$$

$$CNR=\frac{\left|S_{lesion}-S_{psoas}\right|}{\sqrt{{{(SD}_{lesion})}^{2}+{\left({SD}_{psoas}\right)}^{2}}}$$

$$SIR=\frac{S_{lesion}}{S_{psoas}}$$

### 2.6. Statistical Analysis

All statistical analyses were conducted using SPSS software (version 22.0; IBM, Armonk, NY, USA). Measurement data were represented as the mean ± SD. To assess the agreement of the two reviewers for qualitative image quality analyses, Cohen’s weighted kappa statistics were used, with interpretation ranges as follows: 0.00–0.20 indicating poor agreement, 0.21–0.40 fair agreement, 0.41–0.60 moderate agreement, 0.61–0.80 good agreement, and 0.81–1.00 excellent agreement. To assess the agreement of two reviewers for quantitative image quality analysis, intraclass correlation (ICC) was used, with 0.00–0.39 indicating poor reliability, 0.40–0.59 fair reliability, 0.60–0.74 good reliability, and 0.75–1.00 excellent reliability. The Wilcoxon rank-sum test was used to compare the qualitative and quantitative parameters of image quality between MUSE-DWI and DL MUSE-DWI. Statistical significance was set at *p* < 0.05. A Bland–Altman plot was created to assess the concordance of ADC values between MUSE-DWI and DL MUSE-DWI.

## 3. Results

### 3.1. Clinical Characteristics

Patient and tumor characteristics are summarized in Table 1. The mean age of included patients was 67.2 (range: 40–87) and 87.9% were male. Histological diagnosis revealed 49 cases of urothelial carcinoma, 2 cases of paraganglioma, and 1 case each of leiomyoma, inverted urothelial papilloma, papilloma, cystitis cystica, and inflammatory myofibroblastic tumor. One patient did not have a histological confirmation of the mass. The size of the bladder tumors ranged from 2 to 82 mm, with a mean size of 23.8 mm and a SD of 17.8 mm.

### 3.2. Qualitative Image Analysis

**Figure 1:**
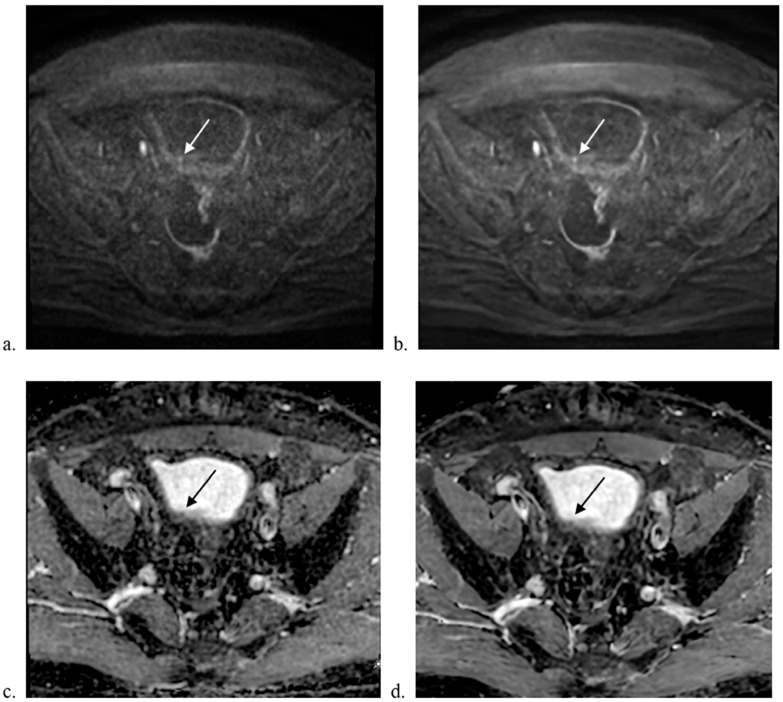
Comparison of MUSE-DWI and DL MUSE-DWI datasets. A 71-year-old male underwent bladder MRI. A tiny papillary lesion was seen at the right posterior wall of the bladder. Transurethral resection confirmed high-grade urothelial carcinoma without involvement of subepithelial connective tissue. Axial images showing (**a**) MUSE-DWI, (**b**) DL MUSE-DWI, (**c**) ADC map generated from MUSE-DWI, and (**d**) ADC map generated from DL MUSE-DWI. The DL MUSE-DWI dataset showed better lesion conspicuity.

**Figure 2:**
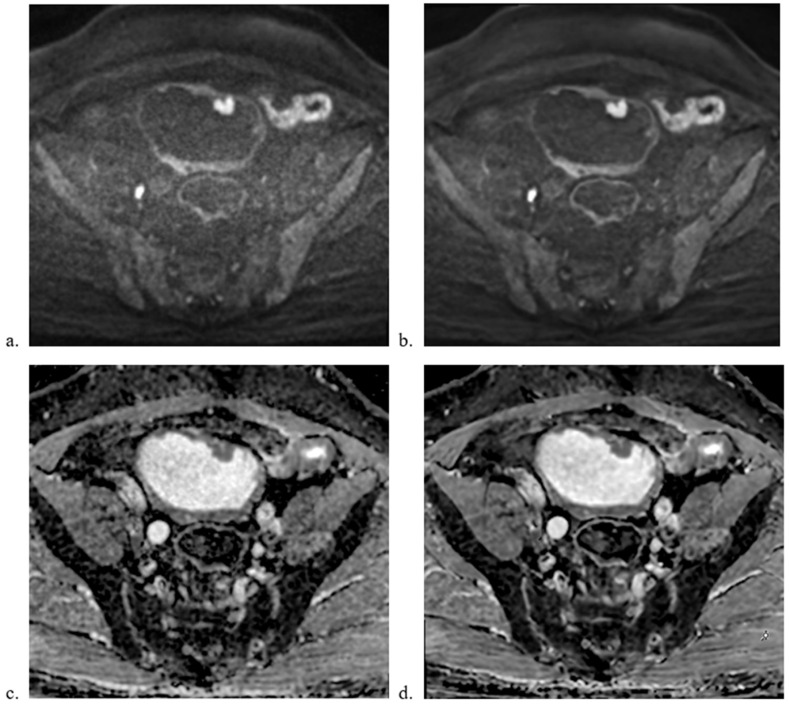
Comparison of MUSE-DWI and ARDL MUSE-DWI datasets. A 65-year-old male underwent bladder MRI. Multiple masses were seen including the two masses at the anterior wall shown in the images. Radical cystectomy confirmed multiple non-invasive papillary urothelial carcinoma. Axial images showing (**a**) MUSE-DWI, (**b**) DL MUSE-DWI, (**c**) ADC map generated from MUSE-DWI, and (**d**) ADC map generated from DL MUSE-DWI. The ARDL dataset showed better sharpness and lesion conspicuity.

Qualitative image analysis revealed that DL MUSE-DWI demonstrated significantly higher sharpness and lesion conspicuity scores than MUSE-DWI for both reviewers. For sharpness, scores were significantly higher for DL MUSE-DWI (2.82 ± 0.54 vs. 3.33 ± 0.61 for reviewer 1; 2.88 ± 0.68 vs. 3.32 ± 0.71 for reviewer 2; *p* < 0.001 for both). Similarly, lesion conspicuity was rated higher for DL MUSE-DWI (3.23 ± 0.6 vs. 3.58 ± 0.65 for reviewer 1; 3.33 ± 0.64 vs. 3.67 ± 0.61 for reviewer 2; *p* < 0.001 for both). In contrast, no significant differences were observed between the two sequences for distortion (*p* = 1 and *p* = 0.317, respectively) or artifacts (*p* = 0.317 for both). The interobserver agreement of the qualitative parameters (sharpness, distortion, artifacts, and lesion conspicuity) is shown in Table 2. Interobserver agreement was good to excellent, with Cohen’s kappa coefficients ranging from 0.61 to 0.86. The results are summarized in Table 3. Representative images are shown in Figure 1 and Figure 2.

### 3.3. Quantitative Image Analysis

**Figure 3:**
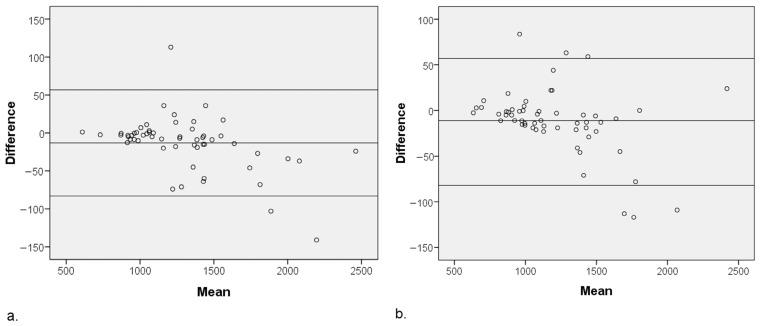
Bland–Altman plot of the ADC values for MUSE-DWI and DL MUSE-DWI techniques in (**a**) reviewer 1 and (**b**) reviewer 2. The central horizontal line indicates the mean difference between the two sequences, while the upper and lower lines represent the limits of agreement (mean ± 1.96 SD). ADC values were significantly different between the two sequences (*p* < 0.001) for both reviewers.

The results of the quantitative image analysis of MUSE-DWI and DL MUSE-DWI are presented in Table 4. DL MUSE-DWI demonstrated significantly higher values for SNR (129.89 ± 104.71 vs. 168.42 ± 145.23 for reviewer 1; 63.63 ± 32.56 vs. 84.15 ± 45.24 for reviewer 2; *p* < 0.001 for both), CNR (6.31 ± 2.13 vs. 6.71 ± 2.66 for reviewer 1, *p* = 0.001; 9.33 ± 4.13 vs. 10.2 ± 4.7 for reviewer 2, *p* < 0.001), and SIR (6.08 ± 2.1 vs. 6.45 ± 2.32 for reviewer 1, *p* < 0.001; 7.01 ± 2.55 vs. 7.25 ± 2.71 for reviewer 2, *p* < 0.001) compared to MUSE-DWI. ADC values were significantly different between the two DWI sequences (1290 ± 361 vs. 1303 ± 378 mm^2^/s for reviewer 1, *p* < 0.001; 1201 ± 352 vs. 1212 ± 367 mm^2^/s for reviewer 2, *p* = 0.002). The Bland–Altman plots of the ADC values for both reviewers are shown in Figure 3.

The interobserver agreement of the qualitative parameters is presented in Table 5. For SNR, the interobserver agreement was poor for both MUSE-DWI (ICC = 0.146, *p* = 0.137) and DL MUSE-DWI (ICC = 0.100, *p* = 0.228). Similarly, the CNR showed poor agreement for both MUSE-DWI (ICC = 0.323, *p* = 0.007) and DL MUSE-DWI (ICC = 0.274, *p* = 0.019). In contrast, interobserver agreement was excellent for the SIR (ICC = 0.866, *p* < 0.001 for MUSE-DWI; ICC = 0.857, *p* < 0.001 for DL MUSE-DWI) and ADC values (ICC = 0.847, *p* < 0.001 for MUSE-DWI; ICC = 0.845, *p* < 0.001 for DL MUSE-DWI).

## 4. Discussion

In this study, we compared the image quality of MUSE-DWI and DL MUSE-DWI using a 3.0 T MRI system. Our findings showed that DL MUSE-DWI had significantly superior image sharpness and lesion conspicuity in the qualitative analysis. Quantitative analysis further supported this, with significantly higher SNR, CNR, and SIR values in DL MUSE-DWI, demonstrating its capacity to improve the overall image quality in bladder imaging.

Previous studies investigating ARDL have reported similar improvements in image quality across various sequences and anatomical regions, including the liver, shoulder, and spine [19,20,21,22,23]. In bladder MRI, one study found that integrating ARDL into standard DWI in bladder MRI significantly improved SNR, CNR, image sharpness, and lesion conspicuity [24]. The improvement in image quality achieved with ARDL is attributable to its inherent ability to reduce noise and suppress ringing artifacts present in imaging data. By directly processing raw complex-valued k-space data, ARDL algorithms effectively suppress artifacts, diminish noise, and enhance image sharpness and clarity [21,25]. Our findings align with those of these studies, reinforcing the potential of ARDLs to improve image quality in bladder MRI, where good spatial resolution and noise reduction are necessary for the detection and characterization of small lesions.

In the qualitative assessment comparing MUSE-DWI with DL MUSE-DWI, significant enhancements were noted in image sharpness and lesion conspicuity. The trained deep convolution neural network integrated within the ARDL pipeline reduces image noise and enhances edge definition, thereby facilitating a clearer delineation of anatomical structures and more conspicuous visualization of lesions [18]. These improvements are critical for clinical diagnostics as they enable more accurate identification and assessment of pathological features. Conversely, no significant differences were observed in image distortion or artifacts. This lack of improvement can be attributed to the inherent sources of these imperfections, such as magnetic field inhomogeneity, patient motion, and fundamental limitations of the EPI sequences used in MUSE-DWI. These factors contribute to complex and multifaceted distortions that are not directly addressed by deep learning-based reconstruction, such as ARDL. Although DL MUSE-DWI does not mitigate distortions and artifacts to a significant extent, its ability to enhance image sharpness and lesion visibility underscores its efficacy in improving the key elements of image quality that are essential for clinical applications.

Interobserver consistency was poor for SNR and CNR measurements, potentially resulting from tumor heterogeneity affecting the drawn ROIs and a lack of consensus regarding ROI placement. This suggests that the selected ROIs may not have fully reflected the entire tumor’s characteristics. However, applying identical ROIs to all sequences within each reviewer’s evaluation minimizes the impact of this limitation on qualitative image assessments. The quantitative values for DL MUSE-DWI were consistently superior to those for MUSE-DWI, regardless of the reviewer. The results of the quantitative analysis supported the findings of the qualitative analysis, highlighting the impact of the ARDL on improving the MUSE-DWI image quality.

A notable observation was the higher ADC values in DL MUSE-DWI than in MUSE-DWI, which is consistent with previous studies involving deep learning-based reconstruction of lung, liver, and breast DWI [26,27,28]. This outcome can be attributed to the improved SNR achieved by DL MUSE-DWI, especially at high b-values, which are crucial for accurate ADC calculations. This improvement in the SNR stems from effectively reducing the rectified noise floor, commonly referred to as the ’Rician noise bias’, which is amplified during multi-nex magnitude averaging, particularly at high b-values where the true signal is weak. Mitigating this noise bias helps avoid underestimation of the ADC in averaged DWI data [29].

We showed that applying ARDL to MUSE-DWI significantly enhanced both the quantitative and qualitative aspects of the resulting images without the typical trade-off of prolonged acquisition times. DL MUSE-DWI not only advanced quantitative measurements but also improved the visual quality of diffusion-weighted images, potentially aiding the early detection and accurate staging of bladder cancer. In the context of VI-RADS assessment, characteristics such as the presence of a stalk, thickened inner layer, and uninterrupted muscular layer signal are key indicators of a low likelihood of muscle invasion [4], which may be more clearly visualized with improved CNR and SNR. This, in turn, may reduce interobserver variability and improve consistency in radiological assessments of bladder cancer. Furthermore, DL MUSE-DWI sequences appear to be a promising option for improving detectability in cases of bladder tumor multiplicity and for enhancing the evaluation of residual and recurrent tumors following treatments such as TURBT. Superior image quality may allow for better differentiation between post-TURBT inflammatory changes and residual or recurrent tumors, ultimately aiding in treatment planning and patient management.

This study has some limitations. First, the retrospective nature of the study and the inclusion of a single-center cohort may have limited the generalizability of our findings. Second, we used a vendor-specific DL algorithm, which may restrict the applicability of our results to other vendor MRI systems and sequences. While this ARDL approach offers benefits such as reduced Gibbs ringing artifacts, diminished noise, and improved image sharpness, it does not fully correct common diffusion-related artifacts—namely EPI distortion, eddy current, and motion effects—which remain important considerations in large-scale DWI studies. Third, owing to the small sample size, we could not subdivide the types of masses and were limited to non-parametric statistical tests, potentially limiting the statistical power of our analysis. Fourth, despite a two-week review interval and a blinded setting, the potential for observer bias remains, as experienced radiologists might still be able to differentiate between MUSE-DWI and DL MUSE-DWI images, potentially influencing the results of qualitative assessments.

## 5. Conclusions

In conclusion, the vendor-specific deep learning algorithm applied to MUSE-DWI resulted in better image quality in terms of sharpness, lesion conspicuity, SNR, CNR, and SIR. Incorporating DL MUSE-DWI into multiparametric bladder MRI protocols may enhance diagnostic accuracy and facilitate better assessment of bladder cancer, potentially aiding clinical decision-making. Further research with a larger population and more diverse tumor subtypes is necessary to validate its clinical utility. Additionally, the observed discrepancies in ADC values between MUSE-DWI and DL MUSE-DWI highlight the need for standardizing ADC measurements across different reconstruction techniques to ensure consistency and reliability in bladder cancer evaluation.

## Figures and Tables

**Table 1:** Clinical characteristics of included patients.

Characteristic	Value
Age, mean (range), years	67.2 (40–87)
Sex (male/female)	50/7
Histologic diagnosis	
Benign lesions	
Leiomyoma	1
Paraganglioma	2
Inverted urothelial papilloma	1
Papilloma	1
Cystitis cystica et glandularis	1
Inflammatory myofibroblastic tumor	1
Malignant lesions	
Urothelial carcinoma	49
No histopathology result	1

**Table 2:** Interobserver consistency of qualitative image quality ratings.

Category	MUSE-DWI		DL MUSE-DWI	
	Kappa	95% CI	Kappa	95% CI
Sharpness	0.64	0.45–0.82	0.62	0.44–0.79
Distortion	0.86	0.72–0.99	0.82	0.67–0.97
Artifacts	0.64	0.32–0.96	0.61	0.3–0.93
Lesion conspicuity	0.75	0.59–0.91	0.73	0.55–0.91

**Table 3:** Comparison of qualitative image quality ratings between MUSE-DWI and DL MUSE-DWI. Values are expressed as mean ± standard deviation.

Index	Reviewer 1			Reviewer 2		
	MUSE-DWI	DL MUSE-DWI	*p* Value	MUSE-DWI	DL MUSE-DWI	*p* Value
Sharpness	2.82 ± 0.54	3.33 ± 0.61	<0.001	2.88 ± 0.68	3.32 ± 0.71	<0.001
Distortion	3.54 ± 0.5	3.54 ± 0.5	1	3.61 ± 0.49	3.63 ± 0.49	0.317
Artifacts	2.98 ± 0.3	3.02 ± 0.48	0.317	3.05 ± 0.35	3.04 ± 0.33	0.317
Lesion conspicuity	3.23 ± 0.6	3.58 ± 0.65	<0.001	3.33 ± 0.64	3.67 ± 0.61	<0.001

**Table 4:** Comparison of quantitative indices of DWI image quality. Values are expressed as mean ± standard deviation.

Index	Reviewer 1			Reviewer 2		
	MUSE-DWI	DL MUSE-DWI	*p* Value	MUSE-DWI	DL MUSE-DWI	*p* Value
SNR	129.89 ±104.71	168.42 ±145.23	<0.001	63.63 ±32.56	84.15 ±45.24	<0.001
CNR	6.31 ± 2.13	6.71 ±2.66	0.001	9.33 ±4.13	10.2 ±4.7	<0.001
SIR	6.08 ±2.1	6.45 ±2.32	<0.001	7.01 ±2.55	7.25 ±2.71	<0.001
ADC value	1290 ± 361	1303 ±378	<0.001	1201 ±352	1212 ±367	0.002

**Table 5:** Interobserver consistency of quantitative indices of DWI quality ratings.

Category	MUSE-DWI		DL MUSE-DWI	
	ICC (95% CI)	*p* Value	ICC (95% CI)	*p* Value
SNR	0.15 (−0.12–0.39)	0.137	0.1 (−0.16–0.35)	0.228
CNR	0.32 (0.07–0.54)	0.007	0.27 (0.02–0.5)	0.019
SIR	0.87 (0.78–0.92)	<0.001	0.86 (0.77–0.91)	<0.001
ADC value	0.85 (0.75–0.91)	<0.001	0.85 (0.75–0.91)	<0.001

## Data Availability

The datasets presented in this article are not readily available because of privacy concerns regarding the clinical data. Requests to access the datasets should be directed to Na Yeon Han.
